# Evidence of Post-COVID-19 Transverse Myelitis Demyelination

**DOI:** 10.7759/cureus.19087

**Published:** 2021-10-27

**Authors:** Sam Kara, Tanner Candelore, Pamela Youssef, Kester Nedd

**Affiliations:** 1 Neurology, Larkin Community Hospital Palm Springs Campus, Miami, USA; 2 Physical Medicine and Rehabilitation, Larkin Community Hospital Palm Springs Campus, Miami, USA

**Keywords:** transverse myelitis, covid-19, acute cervical transverse myelitis, demyelinating diseases, infection

## Abstract

The COVID-19 infection is associated with neurological complications involving both the central and peripheral nervous systems. We present a case of a healthy 36-year-old woman who developed symptoms of transverse myelitis (TM) four weeks following a positive COVID-19 infection. She presented with severe fatigue, bilateral lower extremity ascending tingling, progressive muscle weakness, diminished sensation to pain, temperature and vibration, hyperreflexia, and neurogenic bladder. MRI showed extensive demyelination of the cervical and thoracic spine, and cerebrospinal fluid (CSF) analysis showed mildly elevated protein with normal cell count and no evidence of infection, including negative COVID-19 PCR. The patient was treated with intravenous methylprednisolone dosed daily for five days, and markedly improved and continued to be followed up closely at the office.

## Introduction

COVID-19 infection has claimed the lives of over a million patients worldwide. A range of associated neurological complications, including cerebral thrombosis [1], encephalitis [2], and demyelinating diseases like transverse myelitis (TM), have been described [3,4]. Different TM etiologies have been proposed including autoimmune disorders, paraneoplastic, or infectious/post-infectious disease (such as coronavirus) in which the body’s immune system mistakenly attacks its tissue [5]. TM varies in presentation, is mostly monophasic, and is characterized by acute bilateral ascending or static, loss of sensory, and motor functions. It is associated with significant morbidity and mortality which necessitates increased clinical vigilance, especially considering a possible link during the pandemic [6]. We present a case of post-COVID-19 TM in an immunocompetent patient.

## Case presentation

A 39-year-old female without notable medical, travel, or family history, presented to her work at a correctional facility with a viral syndrome (light headache, rhinorrhea, odynophagia, myalgia, and mild fever) and she subsequently was tested positive for COVID-19. She rested at home, was managed conservatively for her symptoms, re-tested for continuous infection weekly until she tested negative three weeks later. She went back to work and developed feet paresthesias, and subsequently underwent thoracic and lumbar spine computed tomography (CT) scans and non-contrast magnetic resonance imaging (MRI) which showed no pathology per report. Three months later, she presented to our clinic with complaints of numbness, tingling, weakness, and urinary retention. Her neurological examination was notable for incomplete bilateral lower extremity paraparesis with diminished motor strength, and a sensory level at T8-T9 with loss of sensation to lower extremities. Reflexes were increased bilaterally in the lower extremities with positive Babinski bilaterally. A spinal cord pathology secondary to demyelinating disease was considered. The patient was admitted for workup and treatment initiation, and her initial labs revealed a negative COVID-19 and mildly elevated pro-inflammatory markers.

**Figure 1:**
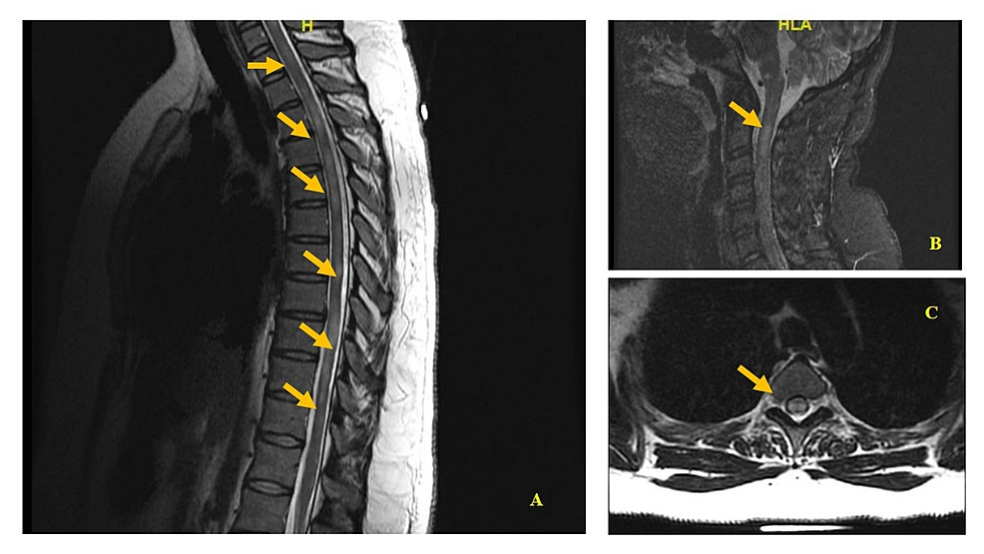
Magnetic resonance imaging of the thoracic spine. (A) and (C) show a plane image of the central spinal cord with a large ill-defined patchy hyperintense signal. (B) Axial T2-weighted cut (arrow) showing a slight expansion of the spinal cord diameter and hyperintense signal without pathologic contrast enhancement. The presence of hyperintensity in the spinal cord could indicate transverse myelitis (arrows).

MRI with gadolinium of cervical spine revealed bilateral extensive cord signal abnormalities, most prominent at the level of C2-C4 and C6-C7 with extensive bilateral hyperintense focus on the medulla (Figures 1A). MRI showed extensive multifocal bright T2 signal lesions involving the thoracic and lumbar spine, some of which demonstrated patchy enhancement (Figure 1B) with no evidence of cord expansion, stenosis, flow voids, foraminal narrowing, and postcontrast sequence did not demonstrate a mass-like enhancement. Thoracic spine MRI with contrast showed multiple longitudinally extensive hyperintense T2-signal abnormality with restricted diffusion and enlargement extending from C5-T12 involving the central gray matter (Figures 1C).

Furthermore, moderately increased T1-signal from T3-T4 to T9-T10 on sagittal imaging and an abnormally diminished gradient-recalled-echo (GRE) signal in the same region was noted. Axial imaging confirmed the abnormal increased T1-signal in this region on sagittal imaging. Postcontrast sequences did not demonstrate a mass-like enhancement. Serology, cerebrospinal fluid (CSF), and immunofixation analysis revealed mild pro-inflammatory markers, and no immunophenotypic evidence of non-Hodgkin’s B-cell lymphoproliferative disorder, T-cells, or acute leukemia were found (Tables 1-5).

**Table 1:** Serum immunofixation workup for transverse myelitis. Results for the serum immunofixation test for the detection and measurement of abnormal proteins are within normal ranges.

Test	Result	Reference
Total protein	6.9 g/dL	5.5–8.7 g/dL
Albumin	3.75 g/dL	3.57–5.42 g/dL
Globulin	3.15 g/dL	2.3–3.5 g/dL
Albumin-globulin ratio	1.2	>1
Alpha 1 globulin	0.33 g/dL	0.19–0.4 g/dL
Alpha 2 globulin	0.77 g/dL	0.45–0.97 g/dL
Beta globulins	0.97 g/dL	0.54–1.07 g/dL
Gamma globulins	1.08 g/dL	0.71–1.54 g/dL
Serum IgA	283.4 mg/dL	70–400 mg/dL
Serum IgG	1052 mg/dL	700–1600 mg/dL
Serum IgM	105 mg/dL	40–230 mg/dL
IgE	103 IU/mL	<100 IU/mL

**Table 2:** Infectious disease workup. Laboratory workup for evidence of infectious diseases most commonly associated with transverse myelitis. CMV: cytomegalovirus, VZV: varicella-zoster virus, EBV: Epstein-Barr virus, HIV: human immunodeficiency virus, Ag: antigen, Ab: antibody, PCR: polymerase chain reaction

Test	Results	Reference
Syphilis serology	Non-reactive	Normal: non-reactive
Lyme total Ab	0.29	<0.90: negative
Chikungunya virus IgG	0.18	0.79 or less
Chikungunya virus IgM	0.58	0.79 or less
SARS-CoV-2 PCR	Negative	Normal: negative
CMV DNA quantification Ab	Negative	Normal: negative
CMV DNA PCR log10	Test not performed	
Enterovirus RNA PCR	Negative	Normal: negative
EBV IgM Ab titer	<0.2	
EBV capsid Ag IgG Ab	>0.8	
EBV nuclear Ag Ab	>8.0	
Herpesvirus 6 IgG Ab	0.26	Below limit of detection
Herpesvirus 6 IgM Ab	<1:20	<1:10 Below limit of detection
HIV 1 and 2 Ag/Ab	Non-reactive	Normal: non-reactive
Influenza A rapid	Negative	Normal: negative
Influenza B rapid	Negative	Normal: negative
Mumps virus IgM Ab	<0.80	0.79 or less
Mycoplasma pneumoniae IgM	Reactive	Normal: non-reactive
Rubeola IgG	1.2	13.4 or less
Rubeola IgM	<0.91	Less than 1.20
VZV IgG Ab	4.8	134.9 or less
VZV IgM Ab	<0.91	Less than 0.91

**Table 3:** Cerebrospinal fluid evaluation. Cerebrospinal fluid evaluation for transverse myelitis etiology. RBC: red blood cells, LDH: lactate dehydrogenase.

Test	Result	Reference
Volume	4 mL	150 mL
Appearance	Clear	Clear
Color	Colorless	Colorless
Supernatant	Clear	Clear
RBC (cells)	2	<10
Total nucleated	1	60–70% lymphocytes; up to 30% monocytes and macrophages; other cells 2% or less.
Glucose (mg/dL)	47	40–85
LDH	10	1/10 of serum level
Total protein (mg/dL)	33	15–45
Prealbumin	8.7	2–7%
Albumin	57.7	56-79%

**Table 4:** Diagnostic workup for autoimmune diseases etiology. Laboratory workup for evidence of autoimmune diseases associated with transverse myelitis. ANA: antinuclear antibodies, SS: Sjogren syndrome, RNP: ribonucleoprotein, Scl: scleroderma, MAG: myelin-associated glycoprotein, GPI: glycoprotein.

Test	Result	Reference
Rheumatoid factor	Negative	Normal: negative
ANA screen	Negative	Normal: negative
JO-1	<0.2	<1
SS-A/Ro Ab	<0.2	<1
SS-B/La Ab	<0.2	<1
Smith Ab	<0.2	19 or less
RNP Ab	<0.2	19 or less
Scl-70 Scleroderma Ab	<0.2	<1
Double-stranded DNA Ab	1.0	Less than 4.9
MAG IgM Ab	<900	Less than 1:1600
Beta-2-GPI IgG Ab	<1.4	Less than 15.0
Beta-2-GPI IgM Ab	1.4	Less than 15.0
Thyroglobulin Ab	<20.0	Less than 20
Thyroid peroxidase Ab	<10	Less than 35
Cardiolipin IgG Ab	<1.6	Less than 40
Cardiolipin IgM Ab	1.6	Less than 40
Complement C3	142	80–178
Complement C4	34.8	12–42

**Table 5:** Cerebrospinal fluid analysis and diagnostic pathology workup. Laboratory and pathological workup for evidence of inflammatory pathogenesis including myelin oligodendrocyte glycoprotein (MOG)-associated disease (MOGAD) and neuromyelitis optica. CSF oligoclonal band assay detected no unique IgG bands.

Test	Result	Reference
MOG fluorescence-activated cell sorting assay	Negative	
Cerebrospinal fluid oligoclonal band assay	Not detected	
Neuromyelitis optica IgG autoantibodies	< .5>	0–3

The patient was treated with methylprednisolone with immediate improvement and was discharged after clinical improvement. One month later, the patient continued to improve, and her cervical-thoracic-lumbar MRI (with and without contrast) revealed moderate resolution of her spinal cord lesions. Partial resolution of cord lesions without expansion was seen in a repeat cervical-thoracic-lumbar MRI with and without contrast.

## Discussion

As the number of COVID-19 infections continues to rise, so is the literature reporting neurological complications [1,7]. Workers providing care for large populations such as correction facilities and hospitals, care for affected patients knowingly or unknowingly and are exposed daily toCOVID-19 [4,8]. We presented a patient who developed TM with longitudinally extensive T2-hyperintensity on cervical-thoracic-lumbar MRI and a punctate focus in the medulla following COVID-19 infection.Low GRE MRI signal intensity along with few RBC cells on CSF analysis is inconsistent with spinal arteriovenous shunts. Brain MRI lacking evidence of encephalitis and clinical picture with no altered mental status or other encephalitic/meningitis symptoms are not consistent with encephalitis further demonstrate a noncerebral etiology.

TM usually involves the spinal cord, but brain involvement has been reported [8-10]. TM has been reported in association with various viral infections such as VZV and HSV-2 in which pathological studies have demonstrated that infections like VZV myelitis, and possibly COVID-19, cause vasculitis, necrosis, and perivascular demyelination causing hemorrhage and necrosis encompassing parenchymal vessels [8-10]. Our patients' workup was negative for infectious or autoimmune etiology (Tables 1-4).

Given that, for example, both influenza virus infection and influenza vaccination have been associated with TM, we hypothesize that our patient may have developed a variant of TM isolated to the spinal cord. As such, her prognosis was poor due to the necrotizing pathology. Additionally, it has been reported to occur in association with different strains of viral infections. In TM, an autoimmune response triggered by infectious, or other unknown agents, causes vascular injury and perivascular demyelination, leading to hemorrhage and necrosis surrounding small parenchymal vessels. Extensive perivascular demyelination, fibrinoid vascular necrosis, broad perivascular mixed inflammatory infiltration, and “ring and ball” hemorrhages are seen in histopathologic specimens. Given that both influenza virus infection and influenza vaccine have been linked to TM, we believe this patient may have developed a spinal-cord-specific version of the disease [11]. As such, her prognosis was poor due to the necrotizing pathology. The pathophysiology of post-COVID-19 ATM is likely similar to that proposed for other post-infectious acute disseminated encephalomyelitis (ADEM). It involves adaptive (e.g., mainly T and B cells, with some plasma cells) and innate immunity (circulating granulocytes, macrophages, monocytes, and activated microglia in the cortex) [12]. Due to cross-reactivity between infectious pathogens and CNS targets, humoral immunity involving antibodies targeting myelin oligodendrocyte glycoprotein (MOG) may be involved.* *Genetic susceptibility based on major histocompatibility complex (MHC) haplotypes may also be involved in the immune response (Table 5).

Infections have been linked to demyelinating diseases of the central nervous system. We presented a case of post-COVID-19 TM with a poor prognosis. More research is needed to identify patients who are vulnerable in a robust and timely manner. 

## Conclusions

The timeline for the COVID-19 symptoms or neurological presentations ranges from days to weeks, thus, rapid and vigilant recognition and management in patients with infectious symptomatology are critical. Although rare, we hypothesize that our patient may have developed TM four weeks post COVID-19. This emphasizes the importance of remaining vigilant to the atypical presentation of COVID-19 and its neurological complications. It is also significant to highlight that young and healthy patients can still develop serious complications, and such a picture emphasizes that more research is needed to identify susceptible individuals.
